# Phytochemical Analysis and Antioxidant Property of Leaf Extracts of *Vitex doniana* and *Mucuna pruriens*

**DOI:** 10.1155/2011/459839

**Published:** 2011-04-18

**Authors:** K. N. Agbafor, N. Nwachukwu

**Affiliations:** ^1^Biochemistry Department, Ebonyi State University, P.M.B. 053 Abakaliki, Nigeria; ^2^Biochemistry Department, Federal University Technology, P.M.B. 1526 Owerri, Nigeria

## Abstract

Oxidative stress and impaired antioxidant system have been implicated in the pathophysiology of diverse disease states. The phytochemical screening and antioxidant property of fresh leaves of *Vitex doniana* and *Mucuna pruriens*, used in the management and treatment of various diseases, were studied. The extracts (ethanol and distilled water) were screened for the presence of phytochemicals, and their inhibition of 2,2-diphenyl-1-picryl-hydrazyl (DPPH) radical was used to evaluate their free radical scavenging activity. Liver levels of malondialdehyde (MDA), superoxide dismutase (SOD), and catalase (CAT) in carbon tetrachloride- (CCl_4_) treated albino rats were also used to assess the antioxidant activity of the extracts. The animals were treated with 250 mg/kg body weight of the extracts for six consecutive days before a single dose (2.5 mL/kg body weight) of CCl_4_. Vitamin C was used as the standard antioxidant. Phytochemical screening revealed the presence of saponins, tannins, anthraquinones, terpenoids, and flavonoids in all the extracts, while alkaloids were detected in extracts of *Vitex doniana* only, and cardiac glycosides occurred in extracts of *Mucuna pruriens* only. All the extracts inhibited DPPH radical in a concentration-dependent manner, water extract of *Vitex doniana* producing highest inhibition which was not significantly different (*P* > .05) from vitamin C. The extracts produced a significant decrease (*P* < .05) in liver MDA, while the levels of SOD and CAT significantly increased (*P* < .05) relative to the positive control. These results are an indication of antioxidant potential of the extracts and may be responsible for some of the therapeutic uses of these plants.

## 1. Introduction

The use of plants in the management and treatment of diseases started with life. In more recent years, with considerable research, it has been found that many plants do indeed have medicinal values [1]. Some medicinal plants used in Nigeria include *Garcina kola*, used in the treatment of asthma, *Carica papaya*, used as a remedy for hypertension, *Ocimum basilicum*, a cure for typhoid fever, and *Cola nitida*, for treatment of pile [2]. *Vitex doniana *(*Verbenaceae*), commonly called black plum, is widely distributed in the eastern and western parts of Nigeria. Various parts of the plant are used by traditional medicine practitioners in Nigeria in the management and treatment of several disorders which include rheumatism, hypertension, cancer, and inflammatory diseases [1]. *Mucuna pruriens *(*Fabaceae*) also called velvet bean is found in Eastern Nigeria, where its seeds are used as soup thickeners. The leaves of *Mucuna pruriens* are used as remedy for various diseases such as diabetes, arthritis, dysentery, and cardiovascular diseases [3]. Phytochemicals are bioactive compounds found in plants that work with nutrients and dietary fibre to protect against diseases. They are nonnutritive compounds (secondary metabolites) that contribute to flavour colour [4, 5]. Many phytochemicals have antioxidant activity and reduce the risk of many diseases, for example, alkyl sulfide (found in onions and garlic), carotenoids (from carrots), and flavonoids (present in fruits and vegetables) [5]. Reactive oxygen-free radicals (ROS) have been implicated in many diseases and in aging process. These free radicals, which cause tissue damage via oxidative stress, are generated by aerobic respiration, inflammation, and lipid peroxidation. Antioxidant systems minimize or prevent deleterious effects of the ROS [6]. 

Lipid peroxidation is an established mechanism of cellular injury and is used as an indicator of oxidative stress. Polyunsaturated fatty acids peroxides generate malondialdehyde (MDA) and 4-hydroxyalkanals upon decomposition [7]. Superoxide dismutase (SOD) decomposes superoxide anion into hydrogen peroxide and oxygen at very high rates. Superoxide radical is involved in diverse physiological and pathophysiological processes [8]. Catalase (CAT) is an antioxidant enzyme ubiquitously present in aerobic cells. It catalyses the decomposition of hydrogen peroxide to water and oxygen. High concentration of hydrogen peroxide is deleterious to cells, and its accumulation causes oxidation of cellular targets such as DNA, proteins, and lipids, leading to mutagenesis and cell death [9]. 

The medicinal applications of *Vitex doniana* and *Mucuna pruriens* have not been given a scientific base. The present study investigates the phytochemical constituents and antioxidant property of the plants.

## 2. Materials and Methods

### 2.1. Collection of Plant Leaves

Fresh leaves of the plants were collected in June, 2010 from a village in Abakaliki of Ebonyi state, Nigeria. They were identified by Professor S.C Onyekwelu of Biology Department, Ebonyi State University, Abakaliki, Nigeria. The leaves were washed, with distilled water, and used immediately.

### 2.2. Extraction of Leaves Material

The extraction methods described by Agbafor [10] were adopted using distilled water and ethanol separately. The local users make use of water or alcoholic drinks for their extractions. After extraction, the solvents were removed using rotary evaporator, to get gel-like extracts.

### 2.3. Phytochemical Screening

The methods of Harbone [11] and Trease and Evans [12] were used to identify the following phytochemicals in the extracts: alkaloids, saponins, tannins, anthraquinones, flavonoids, terpenoids and cardiac glycosides.

### 2.4. Measurement of Antioxidant Property

The antioxidant activity of the extracts was studied in two ways:

(i) Slightly modified method of Brand-Williams et al. [13] using Vitamin C (Emzor Pharmaceutical Industries, Nigeria) as a reference antioxidant. Here, the free radical scavenging properties of the extracts against 2,2-diphenyl-1-picryl hydrazyl (DPPH) radical were measured at 517 nm, as an index to their antioxidant activity. The concentrations of the extracts and Vitamin C used were 1.0, 2.0, 4.0, 6.0, 8.0 and 10.0 mg/mL. Free radical scavenging activity was obtained as;

(1) $$\%\;\text{inhibition}=\;\frac{A_{b}-A_{t}}{A_{b}}\times 100.$$

*A*_*b*_: absorbance of blank, and *A*_*t*_: absorbance of test.

Values were obtained in triplicates.

(ii) Monitoring liver levels of MDA, SOD and CAT in CCl_4_-treated albino rats:

1. *Animals and Handling. *Twenty-eight adult male albino rats, weighing 102–120 g, were brought from the animal house of Biochemistry Department, University of Nigeria, Nsukka, Nigeria. They were placed in seven groups (A–G) of four rats in each group and kept in animals house of Biochemistry Department, Ebonyi State University Abakaliki for seven days to acclimatize. All the rats were allowed free access to feed (rat chaw) and water before and throughout the experiment.
2. *Animal Groups and Treatments. *Solutions of the extracts were made with distilled water. Dose of 250 mg/kg body weight of the extracts and 20 mg/kg body weight of vitamin C (Emzor Pharmaceutical Industries, Nigeria) were given orally to groups A–D and E, respectively, while F and G received distilled water for six consecutive days.
3. *Inducement of Liver Damage. *On the seventh day, groups A–F were treated with a single dose of 2.5 mL/kg body weight of CCl_4_ and olive oil (1 : 1) intraperitoneally. Group G was given distilled water/olive oil (1 : 1).
4. *Collection of Samples from the Animals. *Blood samples were collected from the animals following an overnight fast through cardiac puncture under mild anaesthesia using diethylether. The samples were put into specimen bottles without anticoagulant. Liver was also quickly excised, perfused with cold normal saline, and homogenized in 0.25 M sucrose in phosphate buffer (0.2 M, pH 7.4).

The method of Ohkawa et al. [14] was used to measure the level of MDA. SOD and CAT activities were determined by the methods of Kakkar et al. [15] and Aebi [16], respectively.

### 2.5. Data Analysis

Statistical analysis was done using analysis of variance (ANOVA). Means were compared for significance using Duncan's multiple range test (*P* < .05) [17].

## 3. Results and Discussion

Table 1 shows the results of phytochemical analysis of the four extracts. Saponins, tannins, anthraquinones, terpenoids, and flavonoids were found in all the extracts. Alkaloids were detected in extracts of *Vitex doniana* only, while cardiac glycosides were also present in extracts of *Mucuna pruriens* only. The medicinal values of the plant leaves may be related to their constituent phytochemicals. According to Varadarajan et al. [18], the secondary metabolites (phytochemicals) and other chemical constituents of medicinal plants account for their medicinal value. For example, saponins are glycosides of both triterpene and steroids having hypotensive and cardiodepressant properties [19], while anthraquinones posses astringent, purgative, anti-inflammatory, moderate antitumor, and bactericidal effects [20]. Cardiac glycosides are naturally cardioactive drugs used in the treatment of congestive heart failure and cardiac arrhythmia [21].

Percentage inhibition of DPPH is presented in Table 2. All the extracts inhibited DPPH, indicating their antioxidant activity. The percentage inhibition produced by the water extract of *Vitex doniana* did not show a significant difference (*P* > .05) from those of vitamin C, the standard antioxidant. On the other hand, the inhibitions shown by the other extracts were significantly lower (*P* < .05) than their corresponding values for vitamin C. The inhibition produced by water extract of *Vitex doniana* was higher than that of its ethanol extract while the reverse is the case for *Mucuna pruriens*. All the extracts showed concentration-dependent inhibition.

The DPPH test provides information on the reactivity of compounds with a stable free radical DPPH that gives a strong absorption band at 517 nm in visible region. When the odd electron becomes paired off in the presence of a free radical scavenger the absorption reduces and the DPPH solution is decolorized as the colour changes from deep violet to light yellow. The degree of reduction in absorbance is reflective of the radical scavenging (antioxidant) power of the compound(s) [13]. 

Results of the effect of the extracts on liver concentrations of MDA, SOD, and CAT are presented in Table 3. There was a significant (*P* < .05) increase in MDA levels and decrease in SOD and CAT activities of group F, treated with CCl_4_ only relative to the untreated control group. This reflects hepatotoxicity of CCl_4_, as observed by Singh et al. [22]. The results were reversed on pretreatment with the leaf extracts or vitamin C. The MDA concentration of the pretreated groups was significantly lower (*P* < .05) than the untreated. On the hand, the activities of SOD and CAT were significantly higher (*P* < .05) in the pretreated groups than in the positive control. These observations are indicative of antioxidant property of the extracts. 

Free radical damage and oxidative stress are the major reasons for liver tissue damage. The antioxidant enzymes are the first-line defense against such damage and thus provide protection against the deteriorating outcome [23]. Oxidative injury and lipid peroxidation can be monitored by measuring liver MDA. Lipid peroxidation is regarded as one of the basic mechanisms of tissue damage caused by free radicals [24, 25]. 

The antioxidant activity of the extracts may be attributed to the presence of the identified phytochemicals. Flavonoids and tannins are phenolic compounds, and plant phenolics are a major group of compounds that act as primary antioxidants or free radical scavengers [26]. Similarly, terpenoids, as vitamins, act as regulators of metabolism and play a protective role as antioxidants [27].

The antioxidant property of the extracts may be a strong contributing factor to the applications of the plants in the management and treatment of various diseases. Antioxidants prevent oxidative stress, caused by free radicals, which damage cells and vital biomolecules. They terminate chain reactions triggered by free radicals by removing free radical intermediates and inhibit other oxidation reactions [28]. 

These effects of the extracts on liver MDA, SOD and CAT were maximum in the group treated with water extract of *Vitex doniana*. The effect of water extract of *Vitex doniana* was comparable with that of vitamin C. These observations are consistent with the pattern of inhibition of DPPH by the extracts.

## 4. Conclusion

The presence of the identified phytochemicals makes the leaves pharmacologically active. Their antioxidant activity may be responsible for their usefulness in the management and treatment of various diseases. We are currently studying other possible mechanisms of action of these leaves. Efforts to identify the constituent compounds responsible for this antioxidant activity are also in progress.

## Figures and Tables

**Table 1:** Phytochemical composition of water and ethanol leaf extracts of *Vitex doniana* and *Mucuna pruriens*.

Phytochemical	WVD	EVD	WMP	EMP
Alkaloids	+	+	−	−
Saponins	+	+	+	+
Tannins	+	+	+	+
Anthraquinones	+	+	+	+
Terpenoids	+	+	+	+
Flavonoids	+	+	+	+
Cardiac glycosides	−	−	+	+

+: present, −: absent.

**Table 2:** Percentage inhibition of DPPH by the extracts and vitamins C.

Concentration (mg/mL)	Percentage inhibition (%)				
	WVD	EVD	WMP	EMP	Vit C
1.0	20.42 ± 2.55	14.53 ± 2.66	6.50 ± 1.31	11.32 ± 1.50	28.40 ± 3.72
2.0	31.15 ± 3.60	23.44 ± 1.85	12.61 ± 0.92	17.22 ± 2.11	45.60 ± 2.85
4.0	49.75 ± 2.71	33.25 ± 2.05	18.34 ± 1.65	24.10 ± 1.70	62.55 ± 4.50
6.0	54.20 ± 2.65	39.14 ± 2.11	24.60 ± 2.10	29.25 ± 2.41	74.06 ± 4.48
8.0	76.10 ± 2.90	47.30 ± 2.22	29.20 ± 1.88	36.32 ± 2.11	85.15 ± 3.10
10.0	87.52 ± 3.30	52.22 ± 3.10	32.63 ± 2.20	41.40 ± 1.88	93.20 ± 3.25

Values are mean ± SD, *n* = 3, WVD: water extract of *Vitex doniana, *EVD: ethanol extract of *Vitex doniana,* WMP: water extract of *Mucuna pruriens*, and EMP: ethanol extract of *Mucuna pruriens.*

**Table 3:** Liver MDA, SOD and CAT levels of the animals after treatment.

Group	MDA (nmols/g protein)	SOD (U/mg protein)	CAT (U/mg protein)
A	18.30 ± 1.55	5.72 ± 0.93	148.42 ± 2.60
B	24.63 ± 1.08	6.60 ± 1.22	114.50 ± 2.70
C	28.71 ± 2.17	6.13 ± 0.46	96.11 ± 2.33
D	26.36 ± 2.06	4.50 ± 0.55	115.35 ± 3.66
E	14.15 ± 2.10	8.20 ± 0.78	161.31 ± 2.82
F	48.71 ± 2.50	2.88 ± 0.56	68.33 ± 3.15
G	11.52 ± 2.66	9.36 ± 1.93	177.55 ± 2.43

Values are mean ± SD, *n* = 4.
